# Reflections on implementation of a failure-point–focused outpatient parenteral antimicrobial therapy management program

**DOI:** 10.1017/ash.2022.304

**Published:** 2022-09-22

**Authors:** Alice N. Hemenway, Rebecca L. Stewart

**Affiliations:** 1 Department of Pharmacy Practice, University of Illinois Chicago College of Pharmacy–Rockford Health Sciences Campus, Rockford, Illinois; 2 Department of Infectious Diseases, Mercyhealth Javon Bea Hospital, Rockford, Illinois

## Abstract

Management of outpatient parenteral antimicrobial therapy (OPAT) is complex, and incorporation of a pharmacist can improve outcomes. The creation of new clinical programs is often limited by staffing resources. We describe our collaborative program that utilized a failure-point–focused design process to optimize OPAT activities and management.

Outpatient parenteral antimicrobial therapy (OPAT) is the use of intravenous (IV) antimicrobials in patients outside the hospital setting. The Infectious Diseases Society of America guidelines help clarify who is appropriate for OPAT; however, they do not provide direction on the optimal management team structure.^1^ Improved outcomes have been demonstrated with pharmacist involvement, but these studies are often performed at larger or university-affiliated hospitals.^2–5^

Our facility is a 194-bed community hospital located in a mid-sized city, and an OPAT clinic was established here in 2018. This clinic is currently staffed by 2 infectious disease (ID) physicians, a full-time registered nurse (RN), and a 0.2 full-time equivalent (FTE) pharmacist. In addition to OPAT management, the nurse has multiple job duties and is often used to cover other clinics. The problem of how to optimize care for patients on OPAT given these limited resources led to the creation of an intentional, collaborative OPAT program.

## Methods

The program targets common failure points of OPAT transitions of care and outpatient management to limit the number of patients who may be missed at certain care points. A review of the literature, and an assessment of the clinic’s current processes, revealed the need to focus on 3 failure points: (1) patients who leave the facility on inappropriate IV antimicrobials, without weekly laboratory test orders, or no follow up appointment, (2) delayed assessment of weekly laboratory results, and (3) antimicrobials not stopped as planned and/or peripherally inserted central catheter (PICC) not removed at the end of therapy.

To target failure point 1, the ID physicians created a process in which case management accepts home IV antimicrobial orders solely from themselves, thus creating an unofficial ID consultation requirement. This reduced the inappropriately ordered OPAT, ordering errors, and missed patients. The electronic health record (EHR) system (Epic, Epic Systems, Verona, WI) was optimized through a shared list and the electronic “sticky note” function. Both the list and sticky note allow all members of the OPAT team a quick view of key information for current patients.

For failure point 2, the nurse and pharmacist work together to proactively track the date when laboratory tests are performed and the test results. An Excel database (Microsoft, Redmond, WA) is maintained by the pharmacist and is accessible to all ID team members on an encrypted, shared drive. In addition, each patient on OPAT without laboratory results available through the EHR has a Word document (Microsoft, Redmond, WA) in which laboratory results are entered into a table that can be copied into a note. The nurse or pharmacist review weekly laboratory results as they become available, any abnormal values are discussed with the physician, and a monitoring note is placed in the EHR.

Failure point 3 is targeted by a final follow-up to make sure that antimicrobials are stopped and that the PICC has been removed. Most often, the PICC is removed in the office on the last day of treatment. For lines scheduled to be removed at facilities or by home health agencies at the end of treatment, either the nurse or pharmacists calls to confirm removal.

## Results

The program was assessed using a retrospective cohort study design. After we obtained an exemption from our institutional review board, data were collected for patients who received OPAT from January 1, 2019, to December 31, 2020. During this time, 388 patients receiving OPAT were managed through the ID clinic. These data were further divided into 2 periods: a baseline period 1 (January 1, 2019–October 31, 2019) and an intervention period 2 (November 1, 2020–December 31, 2020). The study included 157 patients (40.5%) in period 1 and 231 patients (59.5%) in period 2. The numbers of male patients, mean ages, and the numbers of patients with either diabetes mellitus or chronic kidney disease were similar between the 2 periods. Data from each period were compared with the Fisher exact test using SPSS version 28 software (IBM, Armonk, NY).

The intervention period had consistently lower rates of hospital readmissions that showed a trend toward significance (10.8% vs 16.6%; *P* = .069). We also detected lower, though not statistically significant, hospital readmissions rates related to an OPAT adverse effect or infection (48% vs 61.5%; *P* = .245). This lack of statistical significance could be due to small case numbers. Previously published studies have reported an average hospitalization rate for patients on OPAT of 20%, with an average of 70% of those hospitalizations associated with OPAT complications.^4,5^ Additional outcome comparisons are listed in Table 1.

**Table 1.** Outcomes

Variable	Total, No. (%)	Period 1 Baseline, No. (%)	Period 2 Intervention, No. (%)	*P* Value
No. of patients	388	157 (40.5)	231 (59.5)	
Hospital readmission	51 (13.1)	26 (16.6)	25 (10.8)	.069
Hospital readmission associated with OPAT or infection	28 (54.9)	16 (61.5)	12 (48.0)	.245
ED visit	51 (13.1)	19 (12.1)	32 (13.9)	.367
ED visit associated with OPAT or infection	20 (39.2)	9 (47.4)	11 (34.4)	.266

Note. ED, emergency department; OPAT, outpatient parenteral antimicrobial therapy.

## Discussion

Our program demonstrates one example of how to optimize OPAT activities within a specialty clinic, and this method has possible application to similar hospitals. Our hospital is a medium-sized, community hospital that provides limited clinical pharmacy services. Like many similar hospitals, our pharmacy FTE is limited. By focusing on failure points, we were able to show the value of a collaborative nurse and pharmacist OPAT management program to our facility leadership. Future research plans include collecting data on specific causes of hospital readmission, linking this information to the failure points targeted and assessing cost savings.

## References

[1] Norris AH , Shrestha NK , Allison GM , et al. 2018 Infectious Diseases Society of America clinical practice guideline for the management of outpatient parenteral antimicrobial therapy. Clin Infect Dis 2019;68:e1–e35. 10.1093/cid/ciy745 30423035

[2] Chung EK , Beeler CB , Muloma EW , et al. Development and implementation of a pharmacist-managed outpatient parenteral antimicrobial therapy program. Am J Health-Syst Pharm 2016;73:e24–e33. 26683676 10.2146/ajhp150201

[3] Shah PJ , Bergman SJ , Graham DR , Glenn S. Monitoring of outpatient parenteral antimicrobial therapy and implementation of clinical pharmacy services at a community hospital infusion unit. J Pharm Pract 2015;28:462–468. 25107418 10.1177/0897190014544786

[4] Means L , Bleasdale S , Sikka M , Gross AE. Predictors of hospital readmission in patients receiving outpatient parenteral antimicrobial therapy. Pharmacotherapy 2016;36:934–939. 27393717 10.1002/phar.1799

[5] Huck D , Ginsberg JP , Gordon SM , et al. Association of laboratory test result availability and rehospitalizations in an outpatient parenteral antimicrobial therapy program. J Antimicrob Chemother 2014;69:228–233. 23887864 10.1093/jac/dkt303

